# Alternative Payment Models and Diabetes Outcomes

**DOI:** 10.1007/s11892-026-01636-3

**Published:** 2026-07-21

**Authors:** Marshall H. Chin

**Affiliations:** Section of General Internal Medicine, Department of Medicine, University of Chicago, 5841 S. Maryland Ave., MC2007, Chicago, IL 60637 USA

**Keywords:** Alternative payment models, Value-based payment, Payment, Diabetes, Quality of care

## Abstract

**Purpose of Review:**

Determine whether and how alternative payment models (APMs) impact diabetes care and outcomes. Explain what APMs are and highlight key principles of effective APMs. Provide a conceptual model for how APMs can support transformation to evidence-based population health delivery models that improve diabetes outcomes.

**Recent Findings:**

Advanced APMs with shared savings or global budgets, along with quality incentives, tended to improve diabetes and composite chronic condition outcomes, and can reduce Medicare Part A hospital and Part B outpatient costs.

**Summary:**

Advanced APMs, particularly models with global budgets that hold health care organizations accountable for total cost of care and quality of care, can improve diabetes outcomes and reduce costs. Careful design and implementation of APMs are essential to provide the appropriate mix of incentives, support, and risk for participants to succeed in APMs and develop effective population health management models.

## Introduction

Diabetes outcomes in the United States are poor and inequitable [1], in part because the payment system is not designed to support and incentivize team-based population health models that provide high-value, cost-efficient care to all persons [2–4]. Nationally diabetes control is suboptimal [5], and many patients experience complications [6].

The predominant fee-for-service (FFS) payment system misallocates resources if the goal is to optimize population health. FFS incentivizes volume of services, not quality of care, and increases costs [7]. Current fee schedules undervalue prevention, primary care, and cognitive services compared to procedures and surgeries [7]. FFS does not encourage health care delivery systems to develop and implement integrated, coordinated care that improves the health of all. Therefore, policymakers are interested in alternative payment models (APMs) that reward health care providers for delivering high-quality, coordinated, cost-efficient care [8, 9].

APMs are heterogeneous and complex. Frequently discussions about APMs lack clarity about what they are and how they are supposed to improve diabetes care and outcomes. This article reviews whether and how APMs impact diabetes care and outcomes, focusing mostly on literature since 2020. It provides a conceptual model for how APMs can support transformation to evidence-based population health delivery models that can improve diabetes outcomes [10]. It updates the most recent review by focusing on advanced APMs beyond pay-for-performance [11]. The article discusses seven APM models to explain key levers for inducing organizational change and care transformation: Comprehensive Primary Care Plus (CPC+) [12, 13], Oregon Coordinated Care Organizations [14], Washington State APM4 [15], Blue Cross Blue Shield (BCBS) Massachusetts Alternative Quality Contract [16, 17], Accountable Care Organization Realizing Equity, Access, and Community Health (ACO REACH) [18], Vermont All-payer ACO [19], and Maryland Total Cost of Care Model [20]. Finally, the paper summarizes key conclusions and pathways forward.

## What is an Alternative Payment Model?

### Definition and Categorization

The biggest, most influential APM demonstrations are led or approved by the United States Department of Health and Human Services Centers for Medicare and Medicaid Services (CMS) / Center for Medicare and Medicaid Innovation (CMMI). CMS / CMMI define APMs as payment models that reward health care providers for delivering high-quality, coordinated, cost-efficient care [8].

The Health Care Payment Learning and Action Network (HCPLAN) APM Framework places APMs into four categories with additional subcategories [21, 22]: (1) FFS – No Link to Quality and Value; (2) FFS – Link to Quality and Value (A: Foundational Payments for Infrastructure and Operations; B: Pay-for-Reporting; C: Pay-for-Performance); (3) APMs Built on FFS Architecture (A: APMs With Shared Savings; B: APMs with Shared Savings and Downside Risk); (4) Population-Based Payment (A: Condition-specific Population-based Payment; B: Comprehensive Population-based Payment; C: Integrated Finance and Delivery System).

Categories 3 and 4 APMs are more likely to impact outcomes than Category 2 pay-for-performance [11]. Therefore, this article focuses on Categories 3 and 4 APMs, which usually include Category 2 quality incentives anyways. In 2024, 44.9% of health care payments in the United States flowed through Category 3 (30.2%) or 4 (14.7%) APMs, with 28.7% in downside risk plans in which participants could lose money [23].

## HCPLAN APM Principles

The HCPLAN APM Framework guide notes 7 principles for effective APMs [21]:

1. Engage patients in APM design and encourage active participation in their care.
2. Payment should drive improvements in care delivery and support innovation.
3. Most national spending should move into Category 3 and 4 shared-risk and population-based payment methods.
4. Value-based incentives should reach the frontline care team.
5. Value-based incentives should be sufficient to drive delivery reform without subjecting providers to risk beyond their financial means and/or clinical scope of care.
6. Value-based payment (VBP) models must take quality and value into account, not just costs.
7. Delivery models (e.g. patient-centered medical homes; accountable care organizations) are not payment models. Different payment models could support the same type of delivery model.

Health equity, improving the health of all persons, is a critical goal. The HCPLAN Health Equity Advisory Team recommended how to design APMs intentionally to advance health equity. It recommended incentives to reduce health disparities [24], clinical and social risk adjustment for payment [25], and payments to community-based organizations to fund collaborative partnerships to address health-related social needs [26]. It also recommended creating incentives for health care delivery organizations to maximize social return on investment rather than short-term financial gain [27]. Social ROI captures the broad range of outcomes that are valuable for a society, including health and economic productivity. Diabetes population health models that improve outcomes for all patients and communities increase social ROI [28].

## Conceptual Model

**Fig. 1:**
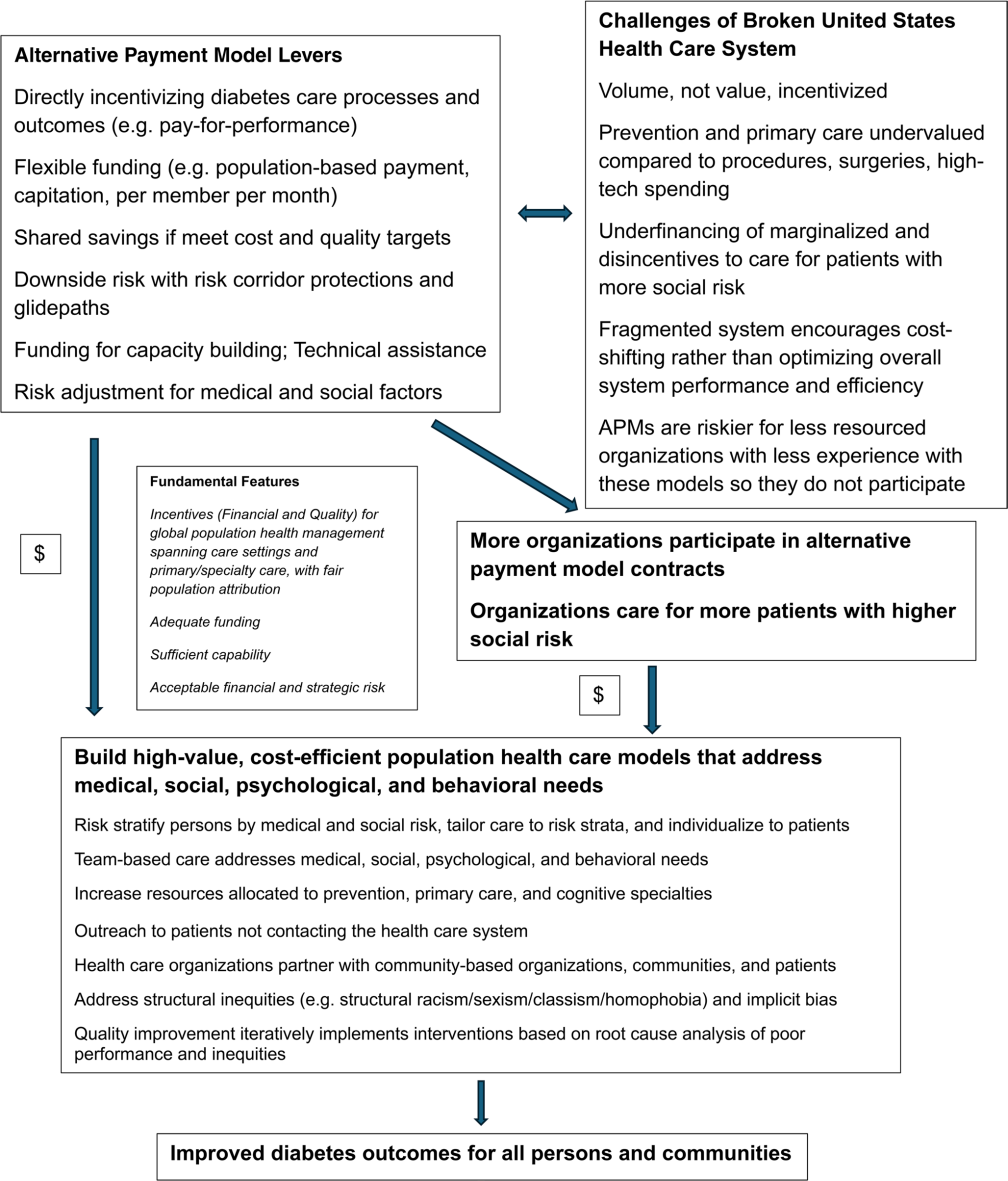
Conceptual model of alternative payment models and diabetes outcomes

To improve diabetes outcomes for all persons and communities, health care delivery organizations should build high-value, cost-efficient population health care delivery models that address medical, social, psychological, and behavioral needs (Fig. 1). The American Diabetes Association Standards of Practice state that effective diabetes population health models feature person-centered team-based care with shared decision making [10]. The evidence-based components of the Chronic Care Model provide a firm foundation for such models [29]: delivery system design with proactive care planning; self-management support for patients; decision support for clinicians; clinical information systems including patient registries; partnerships with community-based organizations; health systems approaches including continuous quality improvement and benchmarking of performance standards, with interventions based on root cause analysis of poor performance and inequities. Care models need to address social drivers of health (SDOH) [30], such as by employing community health workers [31].

Additional key elements of effective population health management include risk stratifying persons by medical and social risk and tailoring care to risk strata [25]; increasing resources allocated to prevention and primary care [7]; and outreach to patients not contacting the health care system. Health care organizations should partner with communities [26], and address structural inequities [32, 33].

Unfortunately, the broken United States health care system largely does not support and incentivize these evidence-based population health models [2]. Volume of services, not value, is incentivized. Prevention and primary care are undervalued compared to procedures and surgeries. The fragmented system encourages cost-shifting rather than optimizing overall system performance and efficiency, and incentivizes cherry picking healthy, well-insured patients to care for rather than patients with higher social risk and those who are uninsured or underinsured.

A critical challenge is that many health systems have their feet in both FFS and value-based payment, and many APMs are built upon an underlying FFS chassis with its accompanying problems. Pay-for-performance programs may reward high quality outpatient diabetes care, but a health system that receives much of its revenue from hospital admissions has limited incentive to invest in and support the most robust population health models that would decrease hospitalizations and their accompanying revenue [34]. In addition, if VBP and APM models account for a relatively small percentage of a health care delivery organization’s revenue or margin, then those models could have limited influence on the organization’s behavior, strategy, and allocation of resources.

To address these problems, advanced APMs provide flexible funding and incentives for health care organizations to build more effective population health care delivery systems. This approach contrasts to creating separate reimbursement codes for community health workers, care coordination, social needs screening, and other factors integral to effective population health models.

Well-designed advanced APMs have several levers that encourage participation and the development and implementation of effective population health models. The most successful APMs have four fundamental features: (1) Incentives (financial and quality) for global population health management spanning care settings and primary/specialty care, with fair population attribution; (2) Adequate funding; (3) Support health organizations to develop sufficient capability to succeed; (4) Acceptable financial and strategic risk for health care organizations.

HCPLAN Categories 2–4 APMs include pay-for-performance incentives that reward some measure of value such as clinical performance metrics. These incentives could be directed at providers, care teams, or clinics. Criteria could be attainment of absolute levels of performance, improvement, performance in relation to a comparison group, or reduction in care disparities and outcomes.

Categories 3–4 APMs provide flexible funding that can be allocated for effective care models. Category 3 APMs can include shared savings and downside risk features. In shared savings, if the health care entity (e.g. health plan) saves money compared to a target benchmarked amount negotiated with the payer (e.g. Medicare), then the entity and Medicare will divide the savings. In downside risk, if the health care entity spent more money than the negotiated target amount, it would lose money. Shared savings arrangements usually require that health care delivery organizations and providers meet quality of care standards to share savings.

Category 4 APMs use population-based payment. Models range from condition-specific population payments (e.g. per member per month payment for primary care) to comprehensive population-based payments that provide a global total payment to cover primary care, specialty care, and hospitalizations. Potential financial gain generally enlarges as risk increases. Comprehensive programs create incentives to coordinate the entire system rather than shift costs to parts of the health care delivery system not covered by the payment program.

Less resourced organizations often do not participate in APMs because they perceive their financial risk would be too high [35]. Some APM programs supply direct funding for infrastructure and capacity building, and provide technical assistance for information technology, financial modeling, and care delivery transformation.

A critical element for advanced APMs is population attribution, the population the health care entity is held accountable for clinical care, health outcomes, and costs. Fair population attribution is essential. Payment models that hold health plans accountable for the health outcomes of a geographic population area rather than only persons seen in the health system create incentives to bring persons into the health care system for needed care. However, those patients should truly be appropriate to be assigned to that particular health system, and data systems must be accurate. Some programs also adjust costs and/or clinical performance targets for the medical and/or social risks of the patients and populations served.

To manage risk and encourage providers and health care entities to participate in APMs, some programs have risk corridors that place limits on the money that can be lost; some have glidepaths to more advanced models (e.g. start with pay-for-reporting a performance metric and then proceed to paying based on performance on that metric).

## Prior Systematic Reviews of APMs and Diabetes Care and Outcomes

The most recent review of APMs and diabetes outcomes is Wang et al.’s 2022 paper in *Health Affairs* [11]. They concluded that HCPLAN Category 3 and 4 APMs yielded greater improvements in diabetes process measures than lower-risk APMs, and Category 4 APMs may improve diabetes outcome measures. Dividing studies by HCPLAN categories, they found that the five Category 2 (Fee-for-Service – Link to Quality and Value) papers had mixed results for processes (e.g. HgbA1c testing). The five Category 3 (APMs Built on FFS Infrastructure (shared savings)) papers showed improved processes such as HgbA1c testing. Overall, the three Category 4 (Population-based Payment) papers showed improved processes (e.g. HgbA1c testing), better or comparable outcomes (A1c, blood pressure, lipids) to a Blue Cross Blue Shield network, and decreased emergency department admission rates for diabetes with complications.

## Methods

### Inclusion Criteria

Papers had to evaluate advanced APMs (Categories 3 and 4) and include traditional diabetes process (e.g. ordering of hemoglobin A1c, ordering of lipid testing, nephropathy testing, dilated eye exam) or outcome (e.g. control of hemoglobin A1c, blood pressure, lipids) metrics and/or measures for which diabetes is a part (e.g. all-condition readmission; unplanned admissions for patients with multiple chronic conditions; timely follow-up after acute exacerbations of chronic conditions). Patients are complex and often have multiple conditions, and clinicians and health care organizations take care of the whole person rather than siloed disease entities. Many frontline health care delivery organizations and APMs focus on composite measures like hospital readmissions and a subset of condition-specific measures that are incentivized in payment programs rather than the hundreds of possible condition-specific measures which would be overwhelming to address. In addition, performance on composite measures are important for improving overall population health, including diabetes care and outcomes. I featured mostly multi-site CMS/CMMI demonstrations rather than single site studies, and highlight APM models that exemplify different combinations and variations of levers.

### Search Strategy

I searched PubMed using search words “alternative payment models” “value-based payment,” and “diabetes,” limiting to United States models and literature 2020 and later.

I reviewed the CMS/CMMI website (https://www.cms.gov) to identify their relevant APM pilots and demonstrations.

I searched every issue of the health policy journal *Health Affairs*, and blogs *Health Affairs Blog*, and *Health Affairs Forefront* 2020 to March 2026 to identify any relevant programs.

I snowball searched, chasing citations and other references and leads. I also checked with experts for their recommendation for other studies, and whether I had missed any key APMs.

## Results

Table 1 shows six key APMs that fit into either Category 3 or 4, depending upon which specific model options a participating health care organization chose.

**Table 1:** Health care payment learning and action network categories 3 and 4 alternative payment models and diabetes outcomes

**Comprehensive Primary Care Plus (CPC+)** **[**12**]**
*Key Features*
2017-2021
Support primary care transformation along tracks that had different care transformation expectations and payment models. Both tracks had care management fees, enhanced payments, data feedback, and group and individualized learning supports. Participants could be in a Medicare Shared Savings Program concurrently, with global incentives to reduce costs and utilization.
Track 1 (1373 practices)
Basic expectations regarding access, teamwork, and quality improvement
Care management fee: $15 per beneficiary per month [PBPM]
Quality and utilization performance payments: $1.25 PBPM each
Median $698,720 per practice over the course of the initiative (9% of practice revenue)
Track 2 (1515 practices)
More advanced expectations for treating patients with complex needs, connecting patients with community resources, and shifting compensation to partial capitation payments.
Care management fee: $28 per beneficiary per month [PBPM]
Quality and utilization performance payments: $2 PBPM each
Reduce fee-for-service payments accompanied by lump-sum payments paid regardless of visits
Median $1,402,232 per practice over the course of the initiative (14% of practice revenue)
*Outcomes*
Compared to matched controls:
No to minimal meaningful changes in claims-based quality-of-care measures: Diabetes process measures (ordering of A1c, eye exam, nephropathy test, composite); Statin for cardiovascular disease patients; Angiotensin-converting enzyme inhibitor or angiotensin receptor blocker for patients with coronary artery disease and diabetes. 1.1 (90% CI 0.6 to 1.6) percent absolute increase in diabetes composite measure.
No meaningful change in unplanned readmissions
Decreases in emergency department visits starting in year 1, and in acute hospitalizations and acute inpatient expenditures in later years.
Associations were more favorable for practices also participating in the Medicare Shared Savings Program and independent practices.
No discernible changes in the total expenditures
Track 1: $1.1 PBPM [90% CI, –$4.3 to $6.6]
Track 2: $1.3 PBPM [90% CI, −$5 to $7.7]
Increased expenditures including enhanced payments
Track 1: $13 PBPM [90% CI, $7 to $18], P < .001
Track 2: $24 PBPM [90% CI, $18 to $31], P < .001
**Oregon Coordinated Care Organizations [** 14 **]**
*Key Features*
Per member per month (PMPM)
Quality Incentive Program
Medicaid 1115 waivers provide flexible funding to address social determinants of health
*Outcomes*
From 2023 to 2024: [14]
HbA1c poor control improved by 6.9%
Adult with diabetes: oral examination increased by 9.0%
COVID pandemic complicates interpretation of results
**Blue Cross Blue Shield Massachusetts Alternative Quality Contract [** 16 **, ** 17 **]**
*Key Features*
Population-based global payment, 2-sided risk, quality-based risk share PMPM, quality PMPM [36]
2023 Pay-for-equity [37–39]
Equity Action Community learning collaborative [40]
*Outcomes*
First 8 years 2009-2016. Compared to privately insured enrollees in control states [16]: (1) Outcome measures for hypertension and control of glycated hemoglobin among enrollees with diabetes improved from 75% in 2009 to 85% in 2016; (2) Increase in the average annual medical spending on claims was $461 less. In the later years of the initial cohorts and across the years of the later-entry cohorts, the savings on claims exceeded incentive payments, which included quality bonuses and providers’ share of the savings below spending targets.
2009-2012. In unadjusted analyses, improved aggregate outcomes measures composite (HgbA1c < 9%, LDL cholesterol < 100 mg/dl, blood pressure control < 140/80 mm Hg for persons with diabetes; LDL cholesterol in patients with coronary artery disease; blood pressure < 140/80 mm Hg for patients with hypertension) [17]
Lower-socioeconomic-status enrollees: 63.6% to 73.8%
Higher-socioeconomic-status enrollees: 65.3% to 76.0%
Slope of improvement greater compared to national and New England Healthcare Effectiveness Data and Information Set (HEDIS) averages
**ACO REACH**
*Key Features*
Updated prior models by [41]: (1) Require health equity plan and collection of demographic and social determinants of health data, and create financial incentives to care for populations with higher social risk [62]; (2) Promote provider leadership and governance; (3) Protect beneficiaries and the Model with more participant vetting, monitoring, and greater transparency.
*Payment*
Capitated payment for certain Part A and B services covered under the fee-for-service (FFS) program of original Medicare provided by Participant Providers and those Preferred Providers who elect to participate in the payment mechanism(s).
*Risk Arrangement and Capitation Payment Mechanisms*
Global Option (100% Shared Savings/Shared Losses)
Total Care Capitation: Reflects the estimated total cost of care for Medicare Part A and B services provided by the Participant Providers and Preferred Providers to the REACH ACO’s aligned population.
Or Primary Care Capitation: Reflects cost of Medicare Part A and B primary care services provided to aligned beneficiaries by certain Participant Providers and Preferred Providers.
Professional Option (50% Shared Savings/Shared Losses)
Primary Care Capitation
*Quality Withholds*
In Program Year (PY) 2024, 2% of a REACH ACO’s Financial Benchmark (the Quality Withhold) was held “at risk,” and 5% is being withheld PY 2026 [42]. A REACH ACO can earn part or all of it back, depending on how well it does on the Quality Measures and other related adjustments.
*Risk Corridors*
PY 2025 Policy: The 1st risk corridor in the Global risk option (100%) was set so CMS did not share in savings until a REACH ACO had generated savings greater than 25% of their benchmark.
PY 2026 Policy: CMS narrowed the 1st risk corridor to be 10% (instead of 25%) for REACH ACOs in the Global risk option (100% risk), so that savings and losses above 10% are shared with CMS [42].
*Outcomes*
Using public performance data used for quality measurement rather than evaluation, compared to controls from September 2024 to September 2025, participating entities had [18,43,45]
No change in all-condition readmission (15.41% vs. 15.43%)
Improvement in unplanned admissions for patients with multiple chronic conditions (30.60 vs. 33.46 / 100 person-years)
Improvement in timely follow-up after acute exacerbations of chronic conditions (non-emergency department [ED] vs. ED location) (77.14% vs. 74.02%)
4% reduction in Medicare spending 4^th^ quarter 2025 compared to benchmark (preliminary)
**Vermont All-Payer ACO**
*Key Features*
All-payer
3 ACO programs: Medicare, Medicaid, commercial
Risk-based payments from each payer flow through the ACO, which distributes the prospective payments to participating hospital providers based on their attributed patients.
Payments are tied to provider performance on quality (access to primary care; deaths from suicide and drug overdose; reducing chronic disease prevalence and morbidity) and spending (growth in all-payer and Medicare total cost of care per beneficiary) measures.
Three payment mechanisms: 1) Medicare’s prospective monthly all-inclusive population-based payments; 2) Medicaid’s fixed prospective payment; 3) Traditional FFS
*Outcomes [* 19 *]*
Clinical measures 2018 to 2021:
Diabetes A1c poor control (hemoglobin A1c > 9%) improved: 58.02% to 9.98%
Controlling high blood pressure (< 140/90 mm Hg) improved: 68.12% to 71.48%
All-cause unplanned admissions for patients with multiple chronic conditions improved: 63.84% to 31.61%
Net Medicare spending 2018-2022:
After accounting for incentive payments, statistically significant reduction in Medicare spending of $757.67 per beneficiary per year (PBPY) (6.3%), or $185.8 million overall
**Maryland Total Cost of Care Model**
*Key Features*
All-payer hospital global budgets with adjustments for quality (e.g. hospital readmission rates) and performance for total cost of care for Medicare FFS beneficiaries
Maryland Primary Care Program provides additional payments to improve primary care
Regional Partnership Catalyst Grants for hospitals for diabetes prevention and management (including Medicare Diabetes Prevention Program) and behavioral health crises
Episode Care Improvement Program to improve quality and efficiency of episodes of care beginning after a hospital discharge
All-payer: Medicare, Medicare, private insurance all pay same rate
*Outcomes [* 20 *]*
Reduced potentially preventable admissions by about 7.2 (90% CI: 9.6, 4.8) admissions per 1,000 beneficiaries or 16.8%
Reduced the probability of 30-day unplanned readmissions by 1.6% points (90% CI 1.9, 1.3) or 8.9%
Increased the probability of follow-up after acute exacerbation of chronic conditions by 1.8 (90% CI: 1.2, 2.5) percentage points or 2.6% in the first four years of the Maryland Total Cost of Care period
Reduced total Medicare Part A and B spending 2019-2022 by 2.1% or $292 (90% confidence interval [CI]: $451, $133)/beneficiary/year

### Comprehensive Primary Care Plus (CPC+)

CPC+’s base program has a Per Beneficiary Per Month (PBPM) care management fee to help support primary care transformation as well as a quality bonus, in total being 9–14% of practice revenue. Participating practices had the option to also participate in a Medicare Shared Savings Program. Compared to matched controls, there were no to minimal meaningful changes in claims-based quality-of-care measures and unplanned readmissions [12]. Emergency department visits started decreasing in year 1, and acute hospitalizations and acute inpatient expenditures decreased in later years. Associations were more favorable for practices also participating in the Medicare Shared Savings Program. Total expenditures did not change and expenditures increased when enhanced payments were included.

### Oregon Coordinated Care Organizations (CCO)

Oregon CCOs featured Per Member Per Month (PMPM) payment, a Quality Incentive Program, and a Medicaid 1115 waiver that allowed flexible funding to address SDOH. With the caveat that the COVID pandemic complicates interpretation of results, from 2023 to 2024 poor control of HbA1c improved by 6.9%, and oral examinations in adults with diabetes increased by 9.0% [14].

### Blue Cross Blue Shield Massachusetts Alternative Quality Contract (BCBSMA AQC) [16, 17]

BCBSMA AQC features population-based global payment, 2-sided risk, quality-based risk share PMPM, quality PMPM [36], and starting in 2023 pay-for-equity and an accompanying Equity Action Community learning collaborative [37–40]. During the first 8 years of the program, compared to privately insured enrollees in control states [16], outcome measures for hypertension and control of glycated hemoglobin among enrollees with diabetes improved from 75% in 2009 to 85% in 2016. The increase in the average annual medical spending on claims was $461 less. In later years, the savings on claims exceeded incentive payments, which included quality bonuses and providers’ share of the savings below spending targets. Composite aggregate diabetes and cardiovascular outcome measures improved in both lower and higher socioeconomic-status enrollees [17].

### ACO REACH

This Medicare model required a health equity plan and collection of demographic and SDOH data, and created financial incentives to care for populations with higher social risk [41]. ACO REACH had capitated payments for certain Part A and B services. A health equity benchmark adjustment for population’s social risk increased spending by $3 per month for each ACO member in the top decile of disadvantage and decreased spending $6 per month for each member in the bottom five deciles. Participants could choose either a global risk arrangement option (100% Shared Savings/Shared Losses) through either Total Care Capitation or Primary Care Capitation, or a Professional Option (50% Shared Savings/Shared Losses) with Primary Care Capitation.

ACO REACH also included a Quality Withhold. In Program Year (PY) 2026, 5% of a REACH ACO’s Financial Benchmark (the Quality Withhold) is held “at risk” [42]. A REACH ACO can earn part or all of it back based on how well it does on the Quality Measures and related adjustments. The Global risk option has risk corridors. In PY 2025 CMS did not share in savings until a REACH ACO had generated savings greater than 25% of their benchmark, and in PY 2026 savings and losses above 10% are shared with CMS [42].

Compared to controls, participating entities had no change in all-condition readmission; improvement in unplanned admissions for patients with multiple chronic conditions (30.60 vs. 33.46 / 100 person-years); improvement in timely follow-up after acute exacerbations of chronic conditions (non-emergency department [ED] vs. ED location) (77.14% vs. 74.02%) [18, 43–45].

### Vermont All-Payer ACO

This all-payer ACO ties risk-based payments to provider performance on quality and spending measures. It is all-payer, in which Medicare, Medicaid, and private insurance all pay the same rate to decrease health care organizations cherry picking patients with higher reimbursing insurance. From 2018 to 2021 [19], poor control of A1c decreased from 58.02% to 9.98%, controlling high blood pressure improved from 68.12% to 71.48%, and all-cause unplanned admissions for patients with multiple chronic conditions decreased from 63.84% to 31.61%. From 2018 to 2022, after accounting for incentive payments, net Medicare spending was reduced $757.67 per beneficiary per year (PBPY) (6.3%), or $185.8 million overall.

### Maryland Total Cost of Care Program

This program has all-payer hospital global budgets with adjustments for quality (e.g. hospital readmission rates) and performance for total cost of care for Medicare FFS beneficiaries. Maryland Total Cost of Care Program reduced potentially preventable admissions by about 7.2 admissions per 1,000 beneficiaries or 16.8%; reduced the probability of 30-day unplanned readmissions by 1.6% points or 8.9%; increased the probability of follow-up after acute exacerbation of chronic conditions by 1.8% points or 2.6%; and reduced total Medicare Part A and B spending by 2.1% or $292/beneficiary/year [20].

### Federal Qualified Health Centers

Table 2 shows studies relating Federal Qualified Health Center involvement in APMs to diabetes outcomes, including the Washington State APM4 demonstration project which improved control of hemoglobin A1c [15]. FQHCs in states with APMs had modestly better blood pressure and glucose control [46].

**Table 2:** Federally qualified health center involvement in alternative payment models and diabetes outcomes

**Washington State APM4 [** 15 **, ** 63 **]**
16 participating federally qualified health centers (FQHCs) 2017-2022
*Key Features*
Shift from FQHC encounter-based Prospective Payment System (bundled rate for each patient visit) to more flexible capitated Per Member Per Month (PMPM) payment
i.e. – Switch from payment for volume of services to volume of assigned Medicaid enrollees
Quality incentive: Quality Improvement Score based on comparison to own baseline, national 50^th^ percentile, and national 90^th^ percentile
*Outcomes*
APM4 compared to control
Quality for patients with diabetes:
Poor HbA1c (> 9%) – Improved by 50%, “high degree of statistical significance”
Blood pressure control (< 140/90 mm Hg) – Improved by 35%, “low” statistical significance
Cost:
$8 PMPM less in participating FQHCs than non-participating FQHCs, but offset by $7.92 PMPM increase compared to prior performance of participating FQHCs (increased managed care enrollment during COVID pandemic)
**National Correlations**
*Markowski et al. JAMA Internal Medicine 2024* [46]
Compare change in performance 2013-2021 of 684 FQHCs in states that adopted APM models that explicitly incentivized quality improvement to centers in states yet to adopt APMs. APM definition had five phenotypes: Medicaid managed care (Managed care organization pays FQHCs; state provides wrap around funding if compensation < prospective payment system), at-cost reimbursement, rebased per visit rate, PMPM payment (prospective PMPM rate based on attributed Medicaid patients, often with quality bonus), and base rate plus quality bonus.
Modest differential improvements in blood glucose control (HbA1c *<* 9%) for individuals with type 2 diabetes (1.02% points; 95% CI, 0.02-2.02) and blood pressure control (< 140/90 mm Hg) for individuals with hypertension (1.02% points; 95% CI, 0.04-2.00)
No evidence of cherry picking patients or limiting care.
*Li et al. Health Affairs 2025* [64]
Value-based payment included pay-for-performance, quality bonuses, payments from risk-pool redistribution, and incentives for Centers for Medicare and Medicaid Services primary care demonstration projects (for example patient-centered medical homes)
Improved blood glucose control (hemoglobin A1c *<* 9%) by 2.12 (95% CI 1.41, 2.82) percentage points and blood pressure control (< 140/90 mm Hg) by 1.43 (95% CI 0.78, 2.09) percentage points pre-pandemic 2014-2019, Uniform Data System. Similar qualitative results during pandemic 2020-2023.
Capitation payment (fees paid by managed care organizations using a preset rate for each enrolled patient assigned to an FQHC during a specified period (usually a month))
Improved blood glucose control by 1.91 (95% CI 0.82, 2.99) percentage points, and blood pressure control by 0.98 (95% CI 0.003, 1.95) percentage points pre-pandemic 2014-2019. Association disappeared during pandemic 2020-2023.

## Conclusions

Advanced APMs with shared savings or global budgets, along with quality incentives, tended to improve diabetes and composite chronic condition outcomes, and bend the cost curve such as reducing Medicare Part A and B costs. However, many health care organizations face competing incentives – a prevented hospitalization may be financially beneficial to them under an advanced APM but represent lost revenue when care is reimbursed by FFS. Not surprisingly provider-owned ACOs tend to have lower overall resource utilization and costs compared to hospital-owned ACOs that profit from payment systems that generate hospital revenue [47].

It remains challenging to get money to flow to care transformation that maximizes population health and emphasizes prevention and primary care. Category 4 APMs (Global budget; Total Cost of Care Models) have strong incentives to decrease total costs and resource utilization, which when linked to clinical performance metrics, supports development of population health care delivery models that aggressively address medical and social needs. For example, Wang et al. examined University of Pittsburgh Medical Center (UPMC) Western Maryland which had created an outpatient Center for Clinical Resources to help care for high-risk patients with chronic disease, within Maryland’s all-payer global budget payment system [48]. For patients referred by primary care physicians, endocrinologists, and inpatient physicians, this center addressed medical and social needs through care coordination, behavioral health case management, nutritional counseling and food delivery, and other integrated services. HbA1c, all-cause hospitalization rates, and diabetes-related emergency department visits all improved. Additional studies should examine transformation of diabetes care models by APMs and how health care organizations integrate their strategic, financial, and clinical operations to implement and achieve these reforms [49]. To facilitate this transformation, payments might be more tightly linked to care improvement activities that lead to improved outcomes [9, 22].

Careful design and implementation of APMs are essential to provide the appropriate mix of incentives, support, and risk for participants [50, 51]. Health care delivery organizations benefit from coaching, technical assistance, and help with infrastructure to succeed in APMs and develop effective population health management models [52]. Ensuring fair risk is critical. Factors include correct population attribution, fair payment rates, risk adjusting payments for medical and social risk, and adapting rules to the relative level of control organizations have over the total cost of care. Mitigating risk and providing technical assistance and capital are especially important for underresourced health care organizations with less APM experience [53, 54]. Glidepaths to participating in progressively advanced APMs are part of the solution.

Co-development of APMs among stakeholders is preferred rather than unilateral imposition by state and federal agencies. Successful APM adoption and implementation do not necessarily occur after APM technical specifications are released. It is difficult to address technical issues wisely without also carefully attending to process, culture, and relationships [55]. Successful APM rollout is analogous to good clinical care, which reflects a symbiotic relationship among partners. Clinicians understand that merely prescribing a medication to a person with diabetes is often insufficient. The process of care, including establishing a relationship and trust, are crucial for developing a treatment plan that will actually be adopted and implemented by the patient, including taking a prescribed medication. Similarly, in qualitative interviews, Medicaid agency leaders and safety-net providers note facilitators to forming Medicaid ACOs include not only flexibility, reduced regulatory complexity, glidepaths with gradually increasing risk, leadership and vision, and data and analytic capability, but also community engagement, trust, and transparency [56].

Within a health care organization, the business, quality improvement, and clinical leadership must align for the money from APMs to be allocated to develop and implement high-value, cost-efficient population health models. The required organizational skills and capabilities are considerable. For example, the Accountable Care Atlas outlines key work area checklists in Governance, Finance, Care Delivery, and Health Information Technology that are necessary for success [57]. In addition, alignment of quality metrics across payers would help increase the magnitude of incentives for health care organizations to change their behavior. Alignment would also reduce administrative burden and make care transformation and participation in APMs logistically more feasible.

The most robust evidence for improved outcomes with reduced savings come from Category 4 total cost of care, global payment APMs. However, in a 2025 national survey of health plans, 55% of respondents thought Category 3B APMs (shared savings and downside risk built on FFS architecture (e.g. episode-based payments for procedures)) will grow the most [23]. Category 3 APMs built on a FFS chassis can be problematic, especially with the challenge of avoiding cost shifting in the overall system. For cost savings, especial attention needs to be given to how primary care and specialty care are integrated in APMs [12]. One proposed solution for these challenging issues is the Global Equity Model that uses global payments, robust primary care, episodic bundled payment and accountability for specialty outcomes and costs, shared governance with the community, and accountability to meaningful outcomes to improve community health and health equity [58, 59].

Ultimately no payment systems, including APMs, can maximize the health of the population unless fundamental underlying problems in the United States health care system are fixed [2], including adequate insurance coverage and financing of marginalized populations [34], and supports and incentives to optimize longterm outcomes of community health and social return on investment [24–27]. The rise of private equity in health is emblematic of a system that too often values cutting costs over health gains [60]. Tools such as distributional cost-effectiveness analysis that incorporate, value, equity, and budgetary concerns could help guide policy decisions [61], but ultimately the nation needs to prioritize health over profit.

Advanced APMs can improve diabetes outcomes and reduce costs, if designed and implemented wisely with the goal of optimizing health for all. Adequate funding, support, and incentives to invest in effective population health models, prevention, and primary care are essential. The advanced APM train is accelerating. We can influence where its money goes, who benefits, and the train’s ultimate destination. APMs should be accountable for what the health care system is supposed to do: improve the health of all.

## Key References

- Centers for Medicare and Medicaid Services. ACO REACH Model Summary of Quality Performance, Financial Performance, and Model Payments. Updated March 11, 2026. Available from: https://www.cms.gov/priorities/innovation/files/aco-reach-qrty-trans-rpt.pdf.
  - ⚬ Latest data for ACO REACH APM.
- Chin MH, Bruch JD, Grogan CM, Huang ES, Kandula NR, Kim DD, Peek ME, Pollack HA. How to Fix a Broken Health Care System: Pathways to Maximize Health and Well-being for All. Diabetes Care. 2026;49(1):44-62.
  - ⚬ Wider health policy context for APMs.
- Colla CH, Muhlestein D, Buntin M, Cheng T, Chin M, Decker W, et al. Commission on Investment Imperatives for a Healthy Nation: Health Financing that Drives Individual and Community Health. National Academies of Sciences, Engineering, and Medicine Discussion Paper. In press.
  - ⚬ Wider health financing context for APMs.
- NORC at the University of Chicago. Evaluation of the Vermont All-Payer Accountable Care Organization Model: 2018–2022. 2024. https://www.cms.gov/priorities/innovation/data-and-reports/2024/vtapm-4th-eval-full-report.
  - ⚬ Evaluation of Vermont All-payer ACO APM.
- Oregon Health Authority. Oregon Health Authority: CCO Metrics 2024 Final Report. Available from: https://www.oregon.gov/oha/HPA/ANALYTICS/CCOMetrics/CCO-Metrics-2024-Final-Report.pdf.
  - ⚬ Evaluation of Oregon CCO.
- Peterson G, Rotter J, Machta R, et al. Evaluation of the Maryland Total Cost of Care Model: Progress Report. Mathematica; 2024. https://www.cms.gov/priorities/innovation/data-and-reports/2024/md-tcoc-1st-progress-rpt.
  - ⚬ Evaluation of Maryland Total Cost of Care APM.
- Singh P, Fu N, Dale S, et al. The Comprehensive Primary Care Plus Model and Health Care Spending, Service Use, and Quality. *JAMA*. Jan 9 2024;331(2):132-146. doi:10.1001/jama.2023.24712.
  - ⚬ Evaluation of CPC+.
- Wang GX, Gauthier R, Gunter KE, et al. Improving Diabetes Care Through Population Health Innovations and Payments: Lessons from Western Maryland. *J Gen Intern Med*. Mar 2023;38(Suppl 1):48-55. doi:10.1007/s11606-022-07918-2.
  - ⚬ Example of how health care delivery organizations have transformed population health management under all-payer global budget APM.
- Washington State Health Care Authority. Alternative Payment Model 4 (APM4) Program Evaluation: Evaluating Cost Effectiveness and Impacts on Patient Outcomes with the APM4 FQHC Value-based Purchasing Model 2022. Available from: https://www.hca.wa.gov/assets/program/leg-report-APM4-evaluation-20230112.pdf.

## Data Availability

No datasets were generated or analysed during the current study.
